# The assessment list for trustworthy artificial intelligence: A review and recommendations

**DOI:** 10.3389/frai.2023.1020592

**Published:** 2023-03-09

**Authors:** Charles Radclyffe, Mafalda Ribeiro, Robert H. Wortham

**Affiliations:** ^1^School of Engineering, University of Bristol, Bristol, United Kingdom; ^2^Centre for Accountable, Responsible, and Transparent AI (ART-AI), University of Bath, Bath, United Kingdom; ^3^Department of Electronic and Electrical Engineering, University of Bath, Bath, United Kingdom

**Keywords:** ethics, Artificial Intelligence (AI), ALTAI, trust, ESG

## Abstract

In July 2020, the European Commission's High-Level Expert Group on AI (HLEG-AI) published the Assessment List for Trustworthy Artificial Intelligence (ALTAI) tool, enabling organizations to perform self-assessments of the fit of their AI systems and surrounding governance to the “7 Principles for Trustworthy AI.” Prior research on ALTAI has focused primarily on specific application areas, but there has yet to be a comprehensive analysis and broader recommendations aimed at proto-regulators and industry practitioners. This paper therefore starts with an overview of this tool, including an assessment of its strengths and limitations. The authors then consider the success by which the ALTAI tool is likely to be of utility to industry in improving understanding of the risks inherent in AI systems and best practices to mitigate such risks. It is highlighted how research and practices from fields such as *Environmental Sustainability, Social Justice, and Corporate Governance* (ESG) can be of benefit for addressing similar challenges in ethical AI development and deployment. Also explored is the extent to which the tool is likely to be successful in being taken up by industry, considering various factors pertaining to its likely adoption. Finally, the authors also propose recommendations applicable internationally to similar bodies to the HLEG-AI regarding the gaps needing to be addressed between high-level principles and practical support for those on the front-line developing or commercializing AI tools. In all, this work provides a comprehensive analysis of the ALTAI tool, as well as recommendations to relevant stakeholders, with the broader aim of promoting more widespread adoption of such a tool in industry.

## 1. Introduction

*Artificial Intelligence* (AI) has long promised benefits to humanity, and in the early twenty-first century these benefits have become tantalizingly close to being realized with the combination of ubiquitous high-performance computing, significant volumes, variety, and velocity of data sets, and advances in applied mathematics. These breakthroughs have shown that *Machine Learning* (ML) techniques, albeit in narrow use cases with somewhat fragility, can out-perform humans at tasks as varied as dog-fighting in fighter jets to distinguishing breeds of cats in online photos (Segarra, 2018; Mizokami, 2020). In parallel with advances in ML, there have also been great steps forward in the field of robotics. In recent years, breakthroughs have compounded and examples such as solving Rubik cubes (Segarra, 2018) with comparable dexterity and superior speed to humans or robotic “dogs” (Stieg, 2020) able to navigate a complex terrain have made it clear that we are now perhaps within a generation from the majority of human physical skills being replicable in a machine. Strategic orchestration using AI is often overshadowed by the sizzle of ML and robotics, but it is important to emphasize that digital process automation, referred to by industry as *Robotic Process Automation* (RPA), is also being adopted at a wholesale rate by diverse organizations ranging from investment banks to medical facilities (Steger, 2020; Willcocks and Craig, 2020). For all the hype of AI today, the reality is that most organizations who seek to promote their AI prowess are actually in the business of merely implementing RPA, but this does not negate the fact that the zeitgeist is very much one of seeking tasks that would be desirable to be performed by machine and innovating in order to do so.

A factor that is all too often overlooked is the immense flow of capital chasing AI opportunities (Arnold, 2020). If capital flows are sufficient, then the promise of long-term financial returns are often self-fulfilling prophecies, despite the temporal mismatch between their application and their sought-after benefits becoming realized. The railway bubble in nineteenth century England was a consequence of the prophecy of mass-transit for all, although this dream took many generations to realize. Equally, the dot-com boom at the turn of the millennium spoke of a future world where everything would be online and digital, but it took a global pandemic to finally realize that vision some two decades hence. Today, capital is flowing toward AI opportunities with equal fervor to the rail and web dreams of the past, but unlike previous “bubbles,” it is flowing at high speed on a global scale (Di Vaio et al., 2023).

In short, recent developments in AI have shown very exciting results with regards to performance and flexibility in the range of applicable domains, and this is being met with significant attention and capital investment. Yet, it is worth considering that with opportunity comes risk, and the full scope of risks from AI are unknown but conceivable. Firstly there are the risks resultant from the regulatory landscape. Regulators might be too eager to limit downside and constrain innovation, or indeed might be too slow which results in an uneven playing field (Marchant, 2011). As an example, the internet “cookie” was invented in 1994, but it wasn't until 2011 that the European Data Protection Supervisor published an opinion that personal data in the EU needed greater protection. This led to the enactment of the General Data Protection Regulation (GDPR) in 2016, which finally came into force 2 years later on May 25th 2018 (European Data Protection Supervisor, 2021). Geoffrey Hinton's discovery that Deep Learning approaches on Big Data sets could lead to more accurate results than alternative approaches on smaller volumes of data marks the genesis of our current AI boom (Hinton et al., 2006). If the speed of regulation matches that of the web, we would only be likely to see AI regulation enforced sometime in the mid 2030s. The delay in regulating personal digital data is predominantly attributed to following a reactive approach to regulation, as opposed to a proactive one.

The second risk is that of the safety issues stemming from the use of AI. In the case of autonomous robotic systems such as self-driving cars, the safety concerns are obvious (Koopman and Wagner, 2017). Faulty mechanics or poor engineering will lead to accident, injury, and tragic loss of life. Similarly, faulty machine vision systems might cause the vehicle to power toward an impact resulting in similarly unfortunate outcomes. We have already seen early examples of such failures (Shepardson, 2017). Safety issues also manifest in unintended consequences stemming from engineering failures in the design of systems. Data sets that contain bias are famous for such outcomes, and have led to discrimination against people of color (Mac, 2021), minority groups (Borgesius, 2018), and women (Dastin, 2018). There is a nascent “ML Ops” community beginning to professionalize the engineering best practice around compute workload allocation, model life-cycle management, and data governance that will, in time, mitigate such risks. However, the risk profile associated with “safety” type issues is still largely invisible to industry and when manifested, does so in sudden and surprising ways (Leslie, 2020).

If the field of ethics is defined with such a scope then it is clear that we stand on the shoulders of the likes of Lewis Mumford when we question not whether technological solutions operate as intended, but whether the consequence of their creation and application was fully foreseen and desired (Mumford, 1934). A maximalist definition of ethics is important as the term “ethical” is seen by lay-people as being the most expansive notion of that which is “good.” A discussion of “ethics” limited to what is legal, or what is safe, by which we include what is non-discriminatory, would be a discussion where outcomes might be seen as “ethical” but in fact have wide ranging social impact in terms of affecting the intersubjective relationships that if analyzed fully might be agreed to being negative in outcome. An example of this would be a content bubble being created on a social network platform. It may be the case that the regulatory regime was appropriate and the solution was compliant, and also one where the engineering controls to eliminate bias, discrimination or other statistically capricious outcomes were well implemented. Yet, this solution may also lead to significant financial returns for the platform or content owner at the expense of the mental health, well-being or relationships of the users (Dang, 2021). Such an example might be “ethical” in the narrow definition, but not in the maximalist one (Gabriel, 2020). This situation highlights a clear danger of labeling something as “ethical,” where the exact opposite might be true.

Against this background we assert that policy makers should decide the extent of desired intervention in the development of industries that use AI, and likewise industry needs to consider the commercial impact of the risk profiles that lie before them. It is in the context of this negotiation between policy and industry that the European Commission's Assessment List for Trustworthy AI (ALTAI) was created, and against the above criteria that the success of it or any other tool must be judged. Since the ALTAI tool has been released, prior literature has focused on examining specific contexts, such as how funders should approach ethical AI, or how the list can be applied in driver-assistance systems (Borg et al., 2021; Gardner et al., 2022). However, there is yet to be a robust analysis and actionable recommendations on ALTAI, particularly aimed at proto-regulators, industry practitioners, and others developing and commercializing AI technologies. Therefore, in this work, the ALTAI tool is reviewed, and an in-depth analysis of strengths and weaknesses of the ALTAI tool is provided. From here, recommendations are proposed, aimed predominantly at proto-regulators and those in industry, to ensure the ALTAI tool is employed successfully and effectively by relevant stakeholders, and these are driving the improvement and encouraging the adoption of this tool.

## 2. Background to ALTAI

The European Commission created the now disbanded "High-Level Expert Group" on Artificial Intelligence (HLEG-AI) in June 2018 to support the implementation of the European Strategy on Artificial Intelligence (European Commission, 2018). The HLEG-AI had 52 members from a mixture of academic, industry, and political backgrounds. Their job was to assess the balance of regulatory oversight required in the European Union (EU) against the impact such oversight might have on the benefits to citizens and the economic benefits to the European economies.

**Figure 1:**
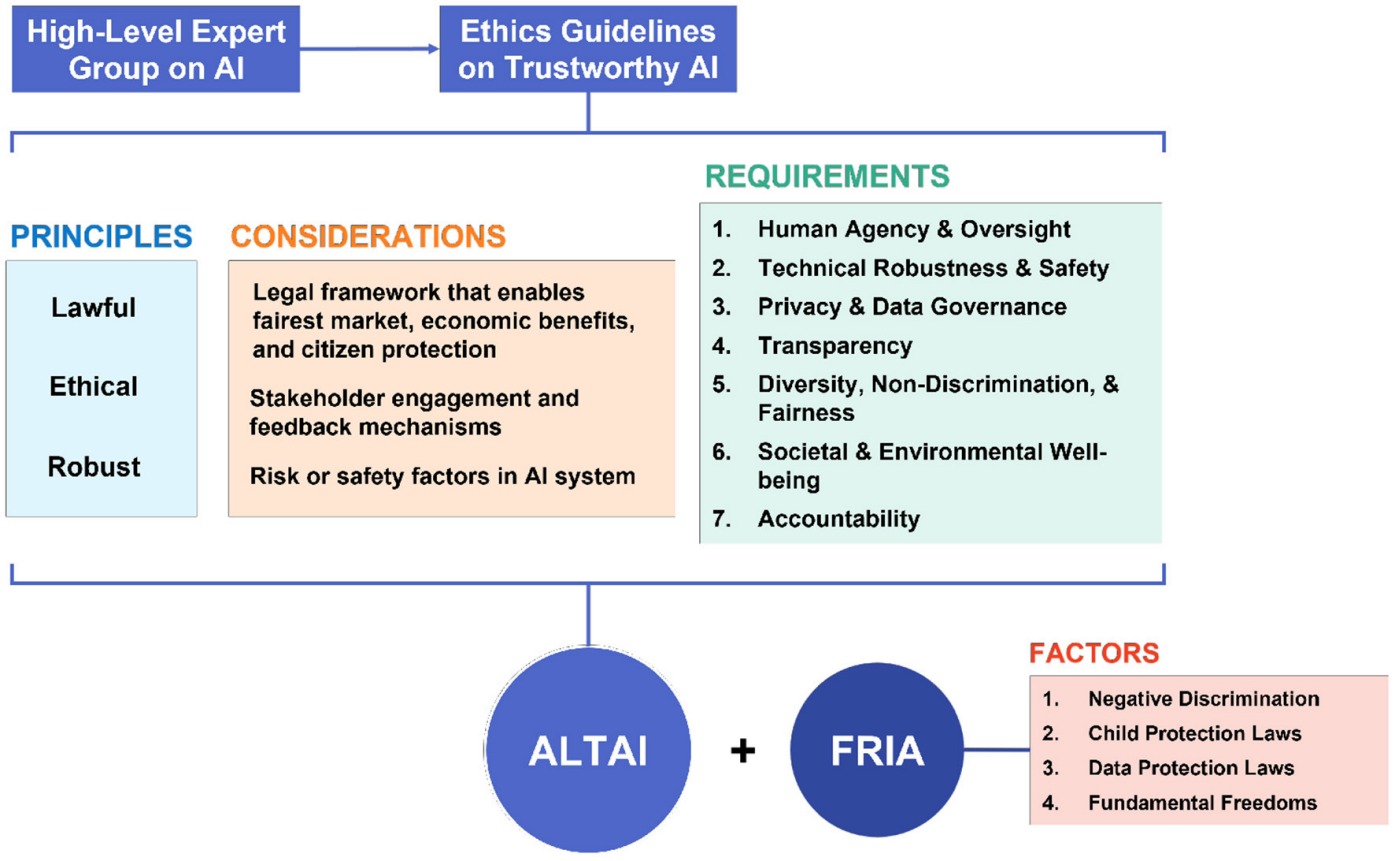
Overview of the different principles, considerations, and requirements shaping the Assessment List for Trustworthy AI (ALTAI) tool. Also included are the factors considered in the Fundamental Rights Impact Assessment (FRIA), which is at present conducted separately.

The process from HLEG-AI, to the different principles and requirements that ultimately shaped the ALTAI tool are summarized in Figure 1. In 2019, the HLEG-AI produced the “Ethics Guidelines for Trustworthy AI” outlining the notion that “trustworthiness” in the EU for AI systems would require components from each of the three pillars of trustworthiness and therefore be lawful, ethical, and robust (High Level Expert Group on AI, 2019). In setting out the three pillars in this way, the HLEG recognized that there are three different domains of governance requiring consideration, and that each are different and require differing management and best practice if trust is to be achieved. It is also worth noting that beyond these domains, trustworthy AI also presents a host of managerial and organizational considerations, which extend to different social and economic perspectives.

The three domains of governance mirror the articulation of risks identified in the introduction above. Legality requires an appropriate legal framework to be adopted, and moreover puts an obligation on industry to engage in public policy debate to seek the appropriate regulatory framework that will enable the fairest market to operate and economic benefits to flow at the same time as giving maximum protection to citizens (Herron, 2020). It is the design of this first domain which is the activity the European Commission is currently engaged with at the time of writing. To be ethical requires engagement with stakeholders and the construction of stakeholder feedback mechanisms (Radclyffe and Nodell, 2020). Finally, robustness requires an identification of the risk or safety factors relevant in the design of a particular AI system, and the design, adoption, and compliance with standards (Hamon et al., 2020).

Extending the three pillars of trustworthiness, the HLEG-AI Guidelines also contain seven key requirements that AI systems should meet in order to be trustworthy, known as the “HLEG-AI Principles.” These are namely:

1. Human agency and oversight
2. Technical robustness and safety
3. Privacy and data governance
4. Transparency
5. Diversity, non-discrimination and fairness
6. Societal and environmental well-being
7. Accountability

Following the publication of the HLEG-AI Principles, the HLEG-AI entered into a consultation process and piloted the guidelines with over 350 stakeholders which ran through to the end of 2019 (European Commission, 2019).

Subsequent to this consultation and pilot, the HLEG-AI published a revised version of the assessment list to the European Commission and in July 2020 launched an online assessment tool, ALTAI (High Level Expert Group on AI, 2020). The ALTAI online tool was developed by Professor Barry O'Sullivan and his team at the Insight Centre for Data Analytics, one of Europe's largest data analytics research organizations located in Cork, Ireland (High Level Expert Group on AI, 2020). The team at Insight have developed the prototype online tool for two reasons: as an implementation of ALTAI, and in order to demonstrate how such an Assessment List such as ALTAI can be of value to industry.

**Figure 2:**
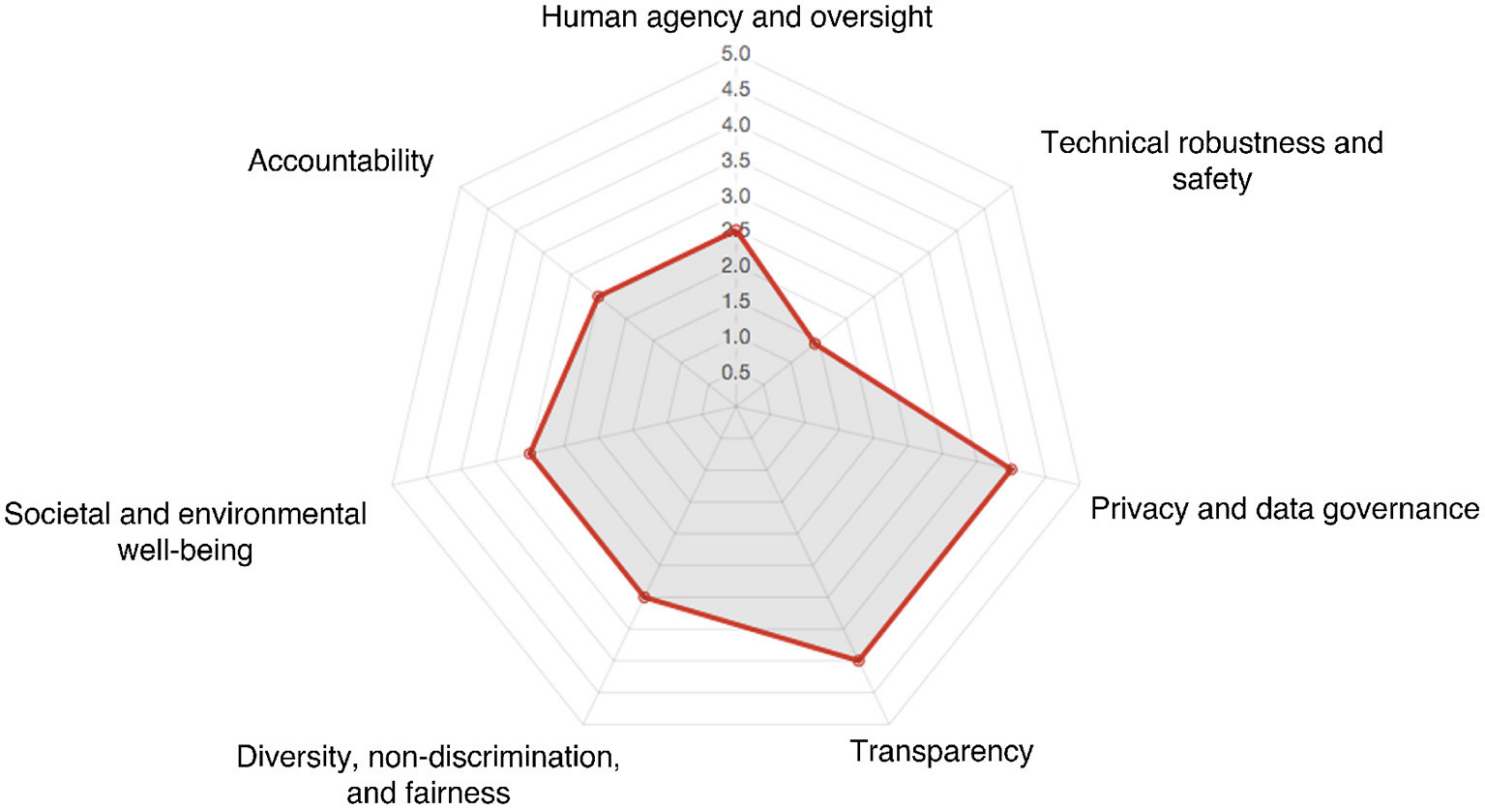
A sample output from the online ALTAI assessment tool (High Level Expert Group on AI, 2020).

ALTAI is implemented as a web-based checklist that organizations are encouraged to complete. Each prompt or question presents either a set options to select from (multiple choice) or allows for text-based input, which is used to verify whether the user has investigated and implemented any measures with regards to the HLEG-AI guidelines outlined above. This results in the creation of a spider-diagram such as the example shown in Figure 2, as well as a list of recommended steps in areas that could be improved. However, it is worth noting that the weighing or contribution of each question to the final scores is not made clear to the user, as well as how any text-based answers are used within the tool.

## 3. Assumptions and prerequisites

In this section we explore and offer comment on the assumptions implicit in the ALTAI tool, together with the prerequisites required for its effective use. Each of the following subsections focusses on one of the axes the ALTAI assessment.

### 3.1. Fundamental rights impact assessment

Before embarking on an assessment, the ALTAI tool recommends to users that they first complete a *Fundamental Rights Impact Assessment (FRIA)*. An FRIA is an exercise which considers an activity in the context of the Charter of Fundamental Rights of the European Union (the Charter), and whether the activity in question is likely to prejudice any fundamental rights as identified in the Charter, or indeed whether it has the effect of promoting them.

A FRIA is commonly conducted in the context of the design and implementation of new legislative instruments, and has recently been completed in anticipation of forthcoming proposed legislation around AI in the EU following the consultation process of AI Regulation which completed earlier in 2020.

The FRIA is however not a common process for private industry to undertake, and therefore given the intention of the ALTAI tool to make it easier for industry to get a sense for what is required of it for “trustworthy AI,” it perhaps might have been expected that an implementation of the FRIA would have been included in the ALTAI itself. Furthermore, given the severity of negative impact that an AI application would have that was found to be in breach of fundamental rights, it would be expected therefore that a failure to conduct an FRIA or a poorly scoring FRIA would have a significant impact on the ALTAI result, which is not the case.

The factors to be considered in an FRIA, prior to application of the ALTAI tool, are outlined in Table 1.

**Table 1:** Overview of factors considered in the FRIA prior to the application of the ALTAI tool.

**Factor**	**Description**
Negative discrimination	An assessment could be made to consider whether the AI system potentially negatively discriminates against people according to protected categories such as gender, ethnicity, or nationality.
Child protection laws	An assessment could be made to assess whether the AI system had the potential to harm child users, or whether adequate monitoring of the well-being of children had been assessed where children were likely impacted by the use of the AI system.
Data protection laws	An assessment could be made as to whether there would be a need for a Data Protection Impact Assessment (DPIA) and an assessment of the necessity and proportionality of the data collection and processing connected with the AI system.
Fundamental freedoms	An assessment could be made as to the potential impact on the citizen's fundamental freedoms such as potential limits on their freedom to express an opinion or be involved in peaceful demonstration.

## 4. The seven principles and their implementation in the ALTAI tool

What follows is an overview of the questions that ALTAI covers, aligned to each of the seven principles proposed previously by the HLEG-AI, see Section 2 above.

### 4.1. Human agency and oversight

The objective for the Human Agency and Oversight questions is to test the subject organization for how maturely they have considered the role of a human-in-command, human-in-the-loop, or human-on-the-loop in the context of the AI system (Boulanin and Verbuggen, 2017; Wachter et al., 2017; Grønsund and Aanestad, 2020). It is held by the HLEG-AI that AI systems should accentuate the flourishing of democracy and support the agency of the individual leading to a more equitable society. Sample questions are:

1. Is the AI System designed to interact, guide or take decisions by human end-users that affect humans or society?
2. Did you ensure a “Stop” button or procedure to safely abort an operation when needed?
3. Did you take any specific oversight and control measures to reflect the self-learning or autonomous nature of the AI system?

### 4.2. Technical robustness and safety

The precautionary principle is in mind when the focus shifts to the requirement that AI systems are developed with a preventative approach to risk (Bourguignon, 2016). This heading suggests that deliberate thought ought to have been given to the likely or reasonably expected harms caused by the AI system and that such potential harms have been minimized through the design process. This is the section with the most questions, which reflects the urgency around risk and safety that is felt by the HLEG-AI. Sample questions are:

1. Did you red-team (Hoffman, 2017) and/or pen-test (Arkin et al., 2005; Parker, 2020) the system?^1^.
2. Did you define risks, risk metrics, and risk levels of the AI system in each specific use-case?
3. Did you put in place a well-defined process to monitor if the AI system is meeting the intended goals?

### 4.3. Privacy and data governance

Given the enormous data volumes required in order to train modern AI systems, should a system be making recommendations on an individual's preference, or predictions of their behavior or characteristics—a level of personal data and personally identifiable information is likely to be required in order for such a system to operate. This creates obligations to the system designer to ensure that privacy by design is engineered into the system, at the same time as ensuring that high standards of data governance are in place. Sample questions are:

1. Did you establish mechanisms that allow flagging issues related to privacy concerning the AI system?
2. Did you consider the privacy and data protection implications of the AI system's non-personal training-data or other processed non-personal data?
3. Did you align the AI system with relevant standards or widely adopted protocols for (daily) data management and governance?

### 4.4. Transparency

The questions on transparency can be grouped under three separate themes. Firstly, the controls around the provenance of data and models in an AI system; secondly, the extent to which the decisions reached by the AI system are explained and the understanding of users assessed, and thirdly, the quality of disclosure and communication to users of the existence and operation of the AI system (Winfield et al., 2021). Sample questions are:

1. Can you trace back which data was used by the AI system to make a certain decision(s) or recommendation(s)?
2. Did you explain the decision(s) of the AI system to the users?
3. In cases of interactive AI systems (e.g., chatbots, robo-lawyers), do you communicate to users that they are interacting with an AI system instead of a human?

### 4.5. Diversity, non-discrimination, and fairness

In the consideration of diversity, non-discrimination, and fairness the HLEG-AI are acutely aware of the potential harms that can be caused from data being collected in an unequal society, and processed by non-diverse teams. The likelihood that this set of eventualities might result in existing inequalities being exacerbated unless it received especial attention would otherwise be too high.

The resultant view is that a requirement for Trustworthy AI is the incorporation of diversity and inclusion throughout the lifecycle of an AI system. Additionally, systems must be user-centric and designed in such a way as that allows all people to adopt them. This is the section with the second most number of questions, which reflects the magnitude of the harms possible through erring on this theme. Sample questions are:

1. Did you establish a strategy or a set of procedures to avoid creating or reinforcing unfair bias in the AI system, both regarding the use of input data as well as for the algorithm design?
2. Did you take the impact of the AI system on the potential end-users and/ or subjects into account?
3. Did you consider diversity and repetitiveness of end-users and/ or subjects in the data?

### 4.6. Societal and environmental well-being

The concern that inappropriate implementation of technology might have potential to disrupt the fabric of society is borne out in the questions in this section. There is a recognition that there is a balance to be struck between benefiting all human beings now, as well as future generations. Again the flourishing of democracy is seen as a concern, and especially the plurality of values and life choices of individuals. Sample questions are:

1. Are there potential negative impacts of the AI system on the environment?
2. Does the AI system impact human work and work arrangements?
3. Could the AI system have a negative impact on society at large or democracy?

### 4.7. Accountability

A common anxiety toward AI and automated systems is the potential for harm if it acts “out-of-control” or indeed the responsibility for control and harm isn't clear because of the complexities in how the AI system is integrated with other AI or non AI systems, or down to the complexity of the supply-chain. Sample questions are:

1. Did you establish a process to discuss and continually monitor and assess the AI system's adherence to this Assessment List for Trustworthy AI?
2. Did you ensure that the AI system can be audited by independent third parties?
3. Did you consider establishing an AI ethics review board or a similar mechanism to discuss the overall accountability and ethics practices, including potential gray areas?

## 5. Analysis of strengths

The ALTAI tool undoubtedly represents a significant step forward in terms of operationalising AI governance. It focuses on the questions that relate to the substantive governance that is in place within organizations. In doing so, it achieves to bring down the conversation about what needs to be done into the detail around process, procedure, and protocols. This is a marked change from the prior focus of the dialogue around “principles.”

There have been many critiques of “principles” based approaches to AI Ethics and AI Governance, yet organizations seemingly turn to this as a first step and too often conclude that this is the only step toward what is needed to be done (Jobin et al., 2019; Whittlestone et al., 2019; Fjeld et al., 2020).

The second strength of the ALTAI tool is that it has proven to be possible to implement the HLEG-AI guidance into an objective tool that can measure the results. Whereas other aspects of *Environmental Sustainability, Social Justice, and Corporate Governance* (ESG) compliance such as carbon emission calculation, or social justice issues such as gender pay gap analysis are more clearly quantitative in their assessment of a standard of “good”; the questions around technology governance that AI Ethics focus on are inherently subjective as such is the nature of ethics—what is right or good to one person or group might not be to another. In this way the tool demonstrates that a review of governance can indeed be constructed such that the result boils down to a numerical value that can be measured. As such, ALTAI is a tremendous leap forward away from much of the debate on what makes for “good” ethics.

The third distinguishing strength of the ALTAI approach is that, with the exception of the fact that the Fundamental Rights Impact Assessment not being built into the tool and scored by it, the scope of the ALTAI tool is sufficiently broad to be said to cover the entire set of issues. The HLEG-AI group previously called for “trustworthy AI” systems to be legal, robust and ethical and the questions assessed cover these three aspects. Where perhaps additional thinking could have been made would be to the nature of the effect on the ecosystem of the subject application—such as whether it in some way diminishes effective competition in the market, or in an other way tilts the deck in favor of its sponsor. Aside from this point, commentators who have criticized the AI ethics domain as being too focused on technical risk will likely be satisfied by the maximalist version of governance promoted by HLEG-AI through ALTAI.

Finally, a consequence of the scaled approach to the presentation of results from ALTAI is that two things are possible. Firstly, an organization can see where its maturity level lies against the reference standard—more on this later. Secondly, whereas most attention is made to the “spider” diagram of Figure 2 the recommendations made by ALTAI regarding the opportunities for improvement are invaluable for organizations seeking to build a road map for the maturity of their governance. A common question heard at events and conferences around the question of AI governance is *where to start?* ALTAI ensures that whatever the maturity of the subject organization or application, the ideas of governance are actionable.

## 6. Assessment of weaknesses

Despite these clear strengths, there are also a number of shortcomings to be reviewed and considered when considering the use of ALTAI as a tool to guide the implementation of governance by industry.

Firstly, what is presented is an objective standard and not one which is relevant to an organization's level of development. A higher standard might be expected from an international corporation with the resources to always ensure high-quality governance, than that of a startup company. The danger with the way that the ALTAI spider diagram presents the results is that it is unappealing for early stage organizations who might score poorly (yet entirely appropriately for their level of sophistication) to present their results publicly, and therefore reducing the opportunity to maximize trust through transparency. While a self-assessment tool is always going to have limited utility to establishing an acceptance of trust, it surely must be an objective to eventually encourage labeling of systems and the quality of their governance—and without some reflection of the expectation of results based on corporate sophistication—this is a major barrier for the tool to be more widely adopted and promoted by startups. We therefore argue that it would be beneficial for corporations to be able to compare their results and performance against others at a similar level of sophistication, or even similar application areas or locations.

The second weakness is that ALTAI does not consider the appropriate level of governance that *ought* to be expected given the firm's maturity of deployment of AI systems. Whereas, our first criticism relates to the inappropriateness of comparing a startup against a multi-national corporation, our second criticism relates to two organizations who are at differing ends of the scale of their maturity in AI adoption. The example here would be a Big Tech company whose AI deployments are absolutely core to their business and have widespread reach. In this case, we would surely insist on there being little excuse for that organization to have anything other than maximum scores in each of the assessment criteria. Contrast this with another example where a global enterprise is making its first tentative steps with experimenting with AI technology. Such steps might include experimenting with datasets or evaluating the possibilities of initiatives to go into limited “proof-of-concept” evaluations. In these cases, whereas we feel *a* level of governance is absolutely appropriate—ALTAI cannot differentiate, and therefore the risk is that there is a disincentive for it to be applied for fear of presenting “low” scores that would otherwise be entirely appropriate.

The third weakness of ALTAI is that there is no consideration given to the relative risk of AI systems in the assessment process. Risk assessment and the granularity of such is a live issue in the European Commission. We have seen many critiques published as to the merits and demerits of a simple—e.g., High/ Low—vs. a more granular multi-tier approach (European Commission, 2020; IEEE & EPPC & IEEE-SA, 2020; Yeung, 2020). Regardless of the stance taken on the desirability for nuance or simplicity, it is clear that the appropriate level of governance concerning an AI system that allocates seats on an airplane is entirely different to that required for a flight-control system based on AI. Risk weighting cannot be taken as a separate exercise and ought be fully embedded in any assessment as to the appropriateness of the level of governance in place.

The fourth weakness of the tool as presented has already been covered in the critique. While it is recognized that a Fundamental Rights Impact Assessment should be conducted prior to running through the ALTAI questionnaire, the consequence of failing such a FRIA would be so significant as to surely warrant reflection in the ALTAI score. This is an urgent oversight that should be addressed in the next revision of the tool.

In addition, some consideration ought to be given to the merits and demerits of the nature of the tool as a *self-assessment* exercise. Whereas, the strength of this approach is that it makes the running of the tool simple, cheap, and easy to implement for subjects; this is a departure to the norms of testing and audit theory that one does not mark one's own homework, and that an independent assessor can offer insights for pragmatic steps and actions for improvement that one simply wouldn't be able to prescribe left to one's own devices. The authors expect that for forthcoming EU regulation around AI to be successful there needs to be an ecosystem of organizations, auditors and rating schemes developed to address the challenges created by industry. Independent auditing is already commonplace in financial reporting and accounting (Zeff, 2004, 2015), and this foundation could be extended for AI and autonomous systems. This is precisely what Carrier (2021) proposes, and this is examined through the lens of different elements contributing to trust, namely predictability, transparency, understanding, control, security, fairness, equity, and morality.

The final criticism, which if addressed would go some way to neutralizing the above concerns, is the imperative for organizations to not solely understand where they sit compared to an arbitrary standard, but how they compare to their peers. An understanding of this factor is what makes other assessment and rating schemes such as credit ratings, academic grades, and car safety schemes successful. Whereas, there may be an objective set of criteria to assess an organization or individual, the most valuable and more importantly *actionable* insight is how the subject compares to their peers. This notion is central to the growing field of ESG, where the initial reaction to any ESG related issue is to first conduct a “materiality” assessment to consider whether the risks being evaluated are sufficiently significant to the organization or industry in focus. From a commercial or organizational perspective, only with this peer comparison can the level of action, and urgency of action, be assessed.

## 7. Calls to proto-regulators

Countries such as the UK and Germany have developed an approach to AI where they have created a governmental body to ensure that adequate promotion is made to industry to adopt technologies, develop skills, and to encourage inward investment in parallel with another body to ensure that the citizenry are adequately protected. In the UK, these two contrasting groups are the Office for AI (OfAI) and the Centre for Data Ethics and Innovation (CDEI) respectively. Whereas, there are undoubtedly certain aspects of AI regulation that are best left to sector regulators such as the Financial Conduct Authority (FCA) and the Prudential Regulation Authority (PRA) in the case of Financial Services in the UK, the authors take the view that bodies such as the CDEI should seek collaboration opportunities with their European counterparts so as to ensure a common high-standard assessment scheme is recognized at an international level rather than see fragmentation across the European ecosystem.

The corollary to this of course is that the “proto-regulator” for AI in each jurisdiction should insist on the uptake and implementation of ALTAI in public body led AI initiatives. In the UK alone there have been several high-profile technology governance scandals in recent times, such as the “mutant algorithm” debacle concerning the allocation of 2020 examination results (Jobin et al., 2019) and the “legacy system” fiasco concerning COVID test results being truncated in the NHS testing system caused by inappropriate information architecture being deployed (Dimakopoulos, 2020; Meaker, 2020).

The UK government was largely successful with its approach to digitisation with web technologies, in main due to the insistence that after a few “quick wins” such as the car safety certificate check (MOT online), that all governmental projects that planned to use the web needed to be run through the central digital team. The same approach ought to be made with AI. A body such as the CDEI ought be put on a statutory footing to look for “quick wins” with AI usage—COVID surely provides many such potential use-cases. Once positive traction has been demonstrated then any future public sector initiative must follow the same course. Given the mission of the European Commission to ensure a high level of trustworthiness of AI which is also shared by the UK Government, further failures of trust as have recently been demonstrated could undermine public trust in AI governance not just in a single jurisdiction, but across European society more widely.

## 8. Calls to industry

An old adage is that trust is hard earned and easily lost. A key structural weakness of the technology industry is that while all actors are keen to promote the benefits of AI technologies, each are well aware that it only takes a small number of mistakes for that hard earned trust to be destroyed to the detriment of all. Therefore, it is in the interest of all actors to establish, protect and build trust through Transparency, Accountability, and Responsibility (Wortham, 2020).

Aside from the clear imperative therefore for industry to support and implement tools such as ALTAI that go as far to highlighting best practice and nudging behavior to greater levels of such best practice, there is also a very clear direction of travel from the European Commission and HLEG-AI toward regulation. Industry are best advised to recognize this and “get with the programme.”

First, the Commission formed the HLEG-AI. Second, the HLEG-AI principles were published. These subsequently turned into guidelines, and now a tool has been released to help implement these guidelines by showcasing maturity against the expected standards. The next step is for regulation to be enacted as surely it will. However, before the use of tools such as ALTAI and the display of their results is mandated, it is in industry's own interest to be on the front foot, gain experience with the application of the tool and ensure adequate feedback is given to the HLEG-AI to ensure subsequent revisions of the ALTAI tool meet the needs of industry. In particular this approach will lead to greater trust being fostered and less controversy being created.

In short, ALTAI has the potential to substantially *de-risk* an organization. If a failure occurs and an organization has scored poorly against the ALTAI score then there is minimal risk of “trust contagion” for the organization. The organization, and wider industry, can say “we knew it was weak, and we are working to fix it.” If a failure occurs and the organization has scored strongly against the ALTAI score then, so long as there is an adequate feedback loop in place to ensure ALTAI is continuously updated, the organization (and wider industry) can say “we did everything reasonably expected of us, and we are working to raise the bar for next time.” Such a regime is unlikely to suffer systemic erosion of trust, and such a regime is one where the “techlash” might indeed dissipate over time. To be sure, a stronger regime of independent third-party audit of AI systems would go further in this regard to creating an infrastructure of trust, but a discussion on the wider apparatus of trust instruments is beyond the scope of this discussion.

## 9. Conclusion

It is the view of the authors that ALTAI is a great step in the right direction. Finally the debate has moved beyond merely a discussion as to what the appropriate AI Ethics principles should be, but rather how to implement them in protocols and practice. Rather than focusing on the use of ALTAI for specific applications, this work reviews the tool and proposes next steps for various stakeholders to effectively leverage it. This work highlights that the scope of ALTAI is sufficiently broad as to neutralize most vectors of concern around the design and application of AI systems, and notwithstanding the absence of integration with the Fundamental Rights Impact Assessment that it recommends.

That said, core weaknesses were also identified, such as the inability of subjects to understand their score relative to that which would be appropriate. It would be preferable, and potentially more motivating, for corporations to be compared either on the basis of sophistication, application domain, or location, so as to be better informed on how they stand in relation to similar corporations and competitors. Indeed, further to this is the fact that the tool does not consider risk-ratings, which from a perspective of citizen transparency is a concern as it acts as a deterrent to adoption which needs to be addressed. These deficiencies can be overcome by greater nuance, but also through peer comparisons, and as such it would be desirable for a ratings approach to be taken, as is the case with credit scores, academic achievement, and car safety assessments. Such a rating ecosystem can achieve the result of helping regulators focus on the most urgent concerns and avoid overreaching, supporting industry with their desire to showcase best practice, and at the same time offer citizens clear transparency as to where the risks and harms might lie.

The authors recommend that public bodies take proactive steps toward encouraging the use of the ALTAI tool, and “proto-regulators” of AI in each jurisdiction need to be given teeth to incentivise such adoption. In the case of industry, appropriate feedback consultation mechanisms are crucial to encourage strong adoption of the tool while being confident that any concerns have been addressed. Organizations ought to prepare for upcoming regulation, guidelines, and best practices, and use ALTAI as an opportunity to promote trust in their AI-based products. In future and beyond these recommendations, it would be vital to address the limitations described earlier in the ALTAI tool, and assessing the different public bodies and corporations engaging with it, as well as comparing the tool with other global efforts for the development of trustworthy AI.

To conclude, ALTAI moves us firmly in the right direction toward the goal of trustworthy governance in AI, but the task lies with industry and public bodies to ensure that the opportunity to use it effectively is seized, and that it forms part of a holistic suite of governance assessment. This work aims to further this goal by offering a detailed review of the ALTAI tool, its strengths and weaknesses, and finally recommendations aimed at proto-regulators and industry practitioners. Such discussions and steps are vital for promoting consumer confidence in AI and the easier adoption of this potentially transformational technology.

## Author contributions

All authors listed have made a substantial, direct, and intellectual contribution to the work and approved it for publication.

## Footnotes

^1^Software penetration testing (pen-testing) refers to authorized attempts to exploit security vulnerabilities and weaknesses in a system to ascertain whether unauthorized access is possible. Red-teaming on the other hand is a technique borne out of military circles where a strategy is tested by way of assumption challenge by a second team, a so-called “Red-Team” who play devil's advocate and are expressly charged with considering counterfactual consequences that the primary strategy might cause. It has been used as a technique in cyber-security best-practice for a long time, and recently has been also identified as a technique that could offer significant value to mitigating risks in other technical domains, such as AI (Brundage et al., 2020).
